# Spatial interpolation techniques comparison and evaluation: The case of ground-based gravity and elevation datasets of the central Main Ethiopian rift

**DOI:** 10.1016/j.heliyon.2024.e32806

**Published:** 2024-06-11

**Authors:** Hailemichael Kebede, Zelalem Demissie, Habte Tadesse, Addis Eshetu

**Affiliations:** a Computational Data Science Program, Addis Ababa University, P.O. Box 1176, Addis Ababa, Ethiopia; b Department of Geology, Wichita State University, Wichita, KS, United States; c Departments of Statistics, Addis Ababa University, Ethiopia; d Institute of Geophysics Space Sciences and Astronomy, Addis Ababa University, Ethiopia

**Keywords:** Kriging, Minimum curvature, Interpolation, Gravity, Elevation

## Abstract

The ground-based gravity data reveals diverse anomaly signatures in areas of the Main Ethiopian rift where active volcanic and tectonic activities are dominant. In such a region ground-based data collection is restricted to existing roads and relies on accessible stations. These resulted in gaps in data, either missing, uneven, or insufficient spatial coverage that must be estimated with proper interpolation techniques. Comparison and evaluations of the spatial interpolation methods that are commonly used in potential field geophysical data analysis were made for the terrestrial gravity and elevation data of the central Main Ethiopian rift. In this research, two widely used interpolation techniques, minimum curvature interpolation, and Ordinary Kriging were compared and assessed. A 10 % hold-out validation was employed, where 90 % of the data points were used to generate interpolated surfaces, which were then evaluated against the remaining 10 %. Following interpolation with each technique, the generated grid was converted into discrete data points (estimated values). These are then compared with the available gravity data, which were deliberately excluded from the gridding process (10 % remaining dataset). The accuracy of each method was assessed by evaluation metrics such as mean value, variance, Mean Absolute Error (MAE), Root Mean Square Error (RMSE), correlation coefficient **(r)**, and R-squared. The results showed that the ordinary Kriging interpolation method outperformed the minimum curvature interpolants for gravity data with all performance metrics, while both interpolants seem to perform equally well for the elevation dataset. Therefore, it is proposed to use the Kriging interpolation method for potential field gravity studies conducted in the central Main Ethiopia rift.

## 1 Introduction

Geophysical and remote sensing studies require regularly gridded anomalies for carrying out data interpretation and analysis. After data preprocessing (cleaning, working on missing data, removing outliers), the next component of data analysis is conducting spatial interpolations which can help to extract proper information about heterogeneous Earth. These techniques are the first we encounter in the potential field and remote sensing data analysis. Applying the methods resulted in regularly gridded anomalies and is used to evaluate physical data in a continuous domain. Therefore, understanding the working principles and various interpolation techniques is required to fill in the missing and un-sampled data locations. Different scholars applied different interpolation techniques both in remote sensing and geophysical studies. In gravity data analysis**,** for instance Refs. [1,2], [[3], [4]], and [5] applied the minimum curvatures gridding to reduce the data gaps and missing information while Universal kriging (UK) methods were used by Ref. [6]. On the other hand and for Digital Elevation Models, some interpolation techniques used were documented in (Erdogan 2009; Salekin et al., 2018; Chaplot et al., 2006). Even though gridding methods produce a rectangular array of regularly spaced values, the gridding methods do not produce the same values at required grid nodes. The reason behind this is the difference in mathematical algorithms used by each method**.** Currently, as the gridding approaches are updated from time to time and guessing the single best approach is hard, various researchers across the globe are still working on comparisons of different interpolations methods for different datasets and study locations ([[7], [8], [9], [10]]; Ajvazi and Czimber 2019; [[11], [12], [13], [14]]). In addition to gravity and elevation datasets, researchers in different areas of the world examined and compared the spatial interpolants to select the best candidate that can be used using various datasets [[7], [8], [9],[11], [12], [13], [14], [15], [16], [17], [18]]. This range of gridding techniques exhibited different performances in estimation. The criteria for the interpolation technique used also involve data statistics, spatial coverage, and the type of quantity examined. This implies that there is no universally accepted “rule of thumb” for choosing the most suitable spatial interpolation techniques for specific scenarios [19]. However, researchers must emphasize selecting the appropriate prediction (estimation) methods while working with potential fields, remote sensing, and various other datasets. The selection of suitable interpolants is based on prediction that aims to minimize the error at an unknown data point [7]. This implies that the usage of the best interpolants will help to reduce uncertainty in datasets related to gravity, magnetic, remote sensing, and other datasets that require gridding for their respective analysis.

This study therefore compares minimum curvature and kriging interpolation techniques, utilizing gravity and elevation data from the central Main Ethiopian rift. The study aids users in selecting the most suitable techniques for gridding potential field datasets, specifically, those related to gravity data and Global Navigation Satellite System (GNSS) measured elevation. Different performance metrics were employed to compare and evaluate the interpolants. These metrics help to quantify the quality and performance of interpolation techniques from different viewpoints and extensive test cases.

## 2 Data and methodology

### 2.1 Gravity datasets

**Fig. 1:**
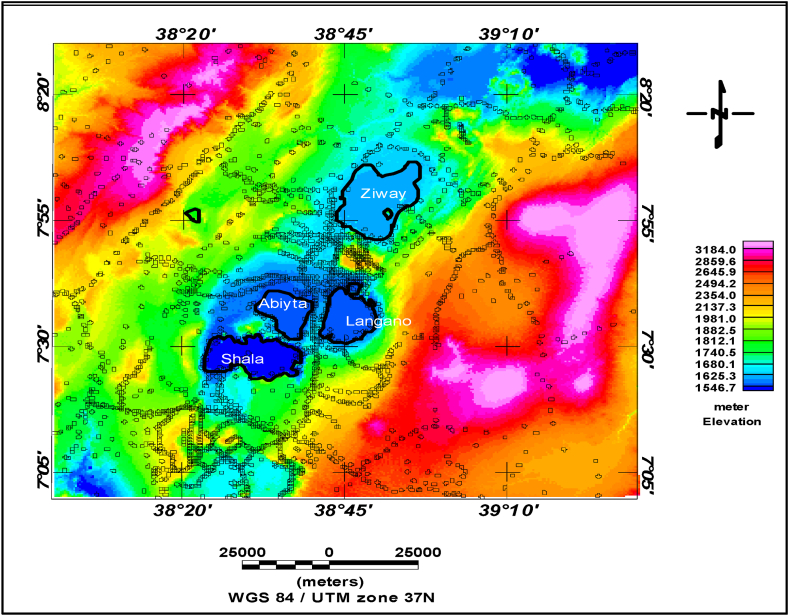
Location of the study area, elevation (color map), and scatter gravity data location map of the central Main Ethiopian rift. (For interpretation of the references to color in this figure legend, the reader is referred to the Web version of this article.)

Ground-based gravity datasets of the region of the central Main Ethiopian rift are used to test the performance of the kriging and minimum curvature interpolants. A total of 3012 ground-based gravity data were used for the analysis, and these datasets are overlaid on top of the elevation surface shown in Fig. 1.

These datasets were sourced from Ph.D. thesis work [20]. The datasets were reprocessed and homogenized with reference to the International Gravity Standardization Network 1971 (IGSN71). To ensure accurate results, the analysis utilized the GRS67 reference system for normal gravity, a reduction density of 2.67 g/cm³ for both Bouguer and terrain corrections, and a free air gradient of 0.3086 mGal/m. Terrain corrections were performed to eliminate the effect of the topographic variations and were calculated using the ASTER Global Digital Elevation Model (30 m resolution). Statistical analyses show that full terrain corrections range from 0.0 to 8.45 mGal with a mean value of 3.09 mGal and a standard deviation of 2.29 mGal. Since the data was collected at different times, error assessment indicates that the resolution of the observed data is estimated at 0.036 mGal. The computed complete Bouguer anomaly data were generated and subsequent data analysis were followed.

### 2.2 Elevations dataset

The elevation data was collected while the ground-based gravity survey was carried out. The dataset consists of approximately 3012 in the central Main Ethiopian rift (Fig. 1) and collected over the past 40 years [1]. It is believed that triangulation and interpolation techniques in elevation determination were used for those data before the use of GNSS techniques. This data is used in the evaluation of the interpolation techniques.

### 2.3 Methodology

The total Bouguer anomaly values used in this research is 3012 and out of this we randomly selected 10 % (counted 301) of the datasets using simple random sampling techniques. The remaining 2711 datasets were gridded with the help of interpolation methods such as minimum curvature and kriging interpolants. The gravity values are estimated on 301 randomly selected locations using the two regular grids, obtained with the interpolation methods under consideration. The interpolation results of the two methods were compared with observed data using commonly used performance metrics measures. The comparison utilized performance metrics such as Mean, variance, correlation, Mean Absolute error (MAE), Root Mean Square Error (RMSE), and R-squared.

#### 2.3.1 Minimum curvature gridding

The minimum curvature gridding is widely used in the earth sciences, especially in potential field geophysics. The interpolated surface generated by Minimum Curvature is analogous to a thin, linearly elastic plate passing through each data value with a minimum amount of bending. Minimum curvature generates the smoothest possible surface while attempting to honor your data as closely as possible (Golden Software Inc. 2002). The algorithm generates the surface that interpolates the available data and solves the modified biharmonic differential equation with tension [21].

(1) $$\left(1-T_{i}\right)\nabla^{2}\left(\nabla^{2}Z\right)-\left(T_{i}\right)\nabla^{2}Z=0$$

Associated to equation. 1, there are three sets of boundary conditions:

$\left(1-T_{b}\right)\frac{\partial^{2}Z}{{\partial n}^{2}}+\left(T_{b}\right)\frac{\partial Z}{\partial n}=0$ and $\frac{\partial \left(\nabla^{2}Z\right)}{\partial n}=0$ on the edges:

$\frac{\partial^{2}Z}{\partial x\partial y}$ at the corners

where:

$\nabla^{2}$ is the Laplacian operator, n is normal to the boundary, $T_{i}$ is the internal tension, $T_{b}$ is the boundary tension and Z is the observed data

#### 2.3.2 Kriging interpolations

Kriging is a stochastic gridding method that uses a linear combination of weights at known locations to estimate the data value of an unknown location[19]. This interpolant is supplemented with a Variogram, from which parameters used for the interpolation are estimated. A variogram is a measure of spatial correlation between two points. With known variograms, weights can change according to the spatial arrangement of the samples. Mathematically, it is given by

(2) $$z_{x}=\sum_{i=1}^{N}\lambda_{i}z_{i}$$

Where, $z_{i}\;\left(i=1,2,.\;.\;.,N\right)$ are observations at N points and the $\lambda_{i}$ are weights. Weights are computed based on distance and also on spatial autocorrelation, which is computed by fitting a semi-variogram to the data. The weight is thus calculated from the semi-variances and the equation of the semi-variance is given by

(3) $$\gamma \left(h\right)=\frac{1}{2N\left(h\right)}\;\left[\sum_{i=1}^{M\left(h\right)}{\left\{z\left(x_{i}\right)-z\left(x_{i}+h\right)\right\}}^{2}\right]$$

Where.

γ(h) is the estimated semivariance or weight at a separation distance or lag h,

$z\left(x_{i}\right)$ is the observed value of the desired variable at some sample location, and

$z\left(x_{i}+h\right)$ is the value of the neighbor separated by h distance.

N(h) is the number of data pairs approximately separated by lag h.

The estimated variogram calculated in equation (Eq. (2)) is an experimental semi-variogram γ(h) calculated from the discrete gravity or elevation datasets. In Kriging, semivariogram is derived from the semivariances. In this process, a function is fitted to the empirical (experimental) variogram to calculate semi-variance at the defined lag distances which can help to determine the weight $\lambda_{i}$. The different variogram functions used are the spherical model, the exponential model, the Gaussian model, the Cubic model, and the Stable model. The default spherical model in Oasis Montaj Geosoft software was used to calculate a theoretical variogram whose coefficients are estimated by a least squares procedure. This spherical variogram is defined

$$\gamma \left(h\right)=C_{o}+C\left[1.5\left(\frac{h}{a}\right)-0.5{\left(\frac{h}{a}\right)}^{3}\right]\;\text{for}\;0<|h|<a$$

(4) $$\gamma \left(h\right)=C_{o}+C\;\text{if}\;\left|\;h\;\right|>a\text{;}$$

$$\gamma \left(h\right)=C_{o}\;\text{if}\;\left|h\right|=0$$

Where:

a is the range-the distance at which the model first flattens out

$C_{o}$ is the nugget effect-the value at which the semi-variogram intercepts the y-value

C is the sill value-the value at which the model first flattens out

h is the interpolation step

**Fig. 2:**
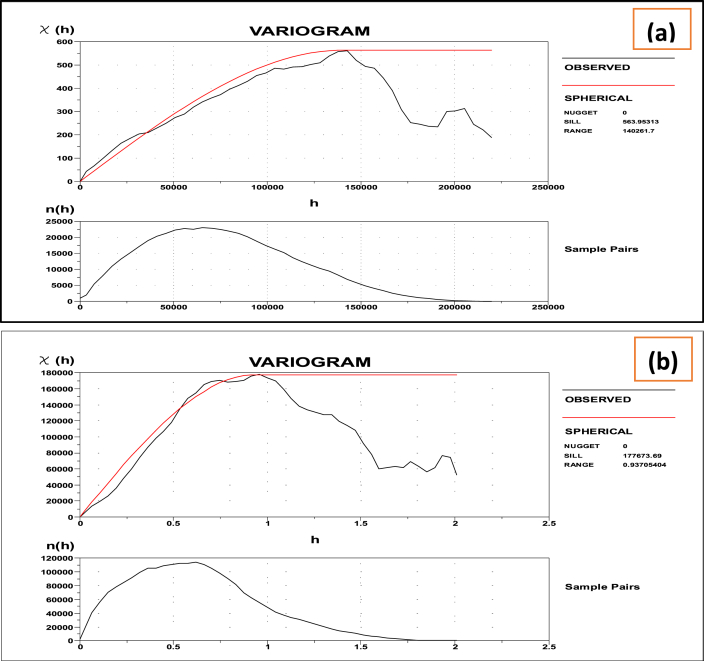
The variogram was estimated with (a) the Gravity and (b) the elevation datasets using the spherical model (red line) fitted to an experimental variogram (black line) and plotted using Oasis Montaj Geosoft software. The subplots shown below each figure indicate the distribution of the number of sample pairs for each lag class. (For interpretation of the references to color in this figure legend, the reader is referred to the Web version of this article.)

Selecting an appropriate model parameter to capture the features of the gravity and elevation data is critical. Here we use the default spherical model of Oasis Montaj Geosoft software to select an estimate to model input parameters. With spherical variogram model, the observed gravity and elevation datasets showed the variogram depicted in Fig. 2 (a and b).

When the separation distance (lag) is zero, the semi-variogram value, also known as the nugget, is zero. This indicates minimal or no measurement errors, or minimal spatial variation at distances less than the sampling interval, or both. Measurement errors arise due to inaccuracies in the measuring devices themselves. Factors such as gravity and elevation can change over different spatial scales. Variations at microscales, smaller than the distances between samples, contribute to the nugget effect. Therefore, it's crucial to understand the different scales of spatial variation before beginning data collection.

### 2.4 Performance metrics

The best interpolation is the one that minimizes the prediction error at KNOWN points. Therefore, the performance (accuracy) of the proposed interpolations method should be made using various performance evaluation techniques. Selection of the most suitable interpolation method out of the two interpolants was made based on commonly used metrics such as Mean, variance, correlation, Mean Absolute error (MAE), Root Mean Square Error (RMSE), and R-squared. The formulas assessing the performance are summarized as follows.

#### 2.4.1 Average (mean)

It is a measure of central tendency and is calculated by adding a group of numbers and then dividing by the count of those numbers (Eq. (5))

(5) $$\text{mean}=\bar{y}=\frac{1}{n}\sum_{i=1}^{n}{\;y}_{i}$$

#### 2.4.2 Mean Absolute Error (MAE)

It calculates the modulus average of the difference between the measured and the estimated values (Eq. (6))

(6) $$\text{MAE}=\frac{1}{n}\sum_{i=1}^{n}|{\;y}_{i}-{\hat{y\;}}_{i}|$$

#### 2.4.3 Root Mean Square Error (RMSE)

It is defined by taking the difference between values observed and predicted by a model (Eq. (7) [22]. The value of the RMSE must tend to 0 and is calculated according to the following relationship

(7) $$\text{the}\;\text{RMSE}=\sqrt{\frac{1}{n}\left(\sum_{i=1}^{n}{\left({\;y}_{i}-{\hat{y\;}}_{i}\right)}^{2}\right)}$$

#### 2.4.4 Correlation coefficient (r)

This metric quantifies the relationship between two variables, serving as a tool to determine the nature of the association and to assess the strength of the relationship. It is governed by

(8) $$r=\frac{n\left(\sum_{i=1}^{n}{\;x}_{i}{\;y}_{i}\right)-\left(\sum_{i=1}^{n}{\;x}_{i}\right)\left(\sum_{i=1}^{n}{\;y}_{i}\right)}{\sqrt{[n\sum_{i=1}^{n}{{\;x}_{i}}^{2}-{\left(\sum_{i=1}^{n}{\;x}_{i}\right)}^{2}]}[n\sum_{i=1}^{n}{{\;y}_{i}}^{2}-{\left(\sum_{i=1}^{n}{\;y}_{i}\right)}^{2}]}$$

The formulas in Eq. (8) provide a value between −1 and 1, where −1 shows negative correlation and +1 shows a positive correlation.

#### 2.4.5 R-squared (coefficient of determination)

It is a statistical measure in a regression model that determines the proportion of variance in the dependent variable that can be explained by the independent variable [23]. It is the goodness of fit.

(9) $$R^{2}=\left(1-\frac{\sum_{i=1}^{n}{\left({\;y}_{i}-{\hat{y\;}}_{i}\right)}^{2}}{\sum_{i=1}^{n}{\left({\;y}_{i}-\;\bar{y}\right)}^{2}}\right)x100\%$$

Where,

$\bar{y}$ is the mean value

${\;y}_{i}$ is the observed data

${\hat{y\;}}_{i}$ is the predicted value from the interpolants

The optimum spatial interpolation method was determined by comparing the metrics values. Here, the calculations determined using MAE and RMSE are expected to be good if the values are smaller—however, the value of $R^{2}$ should approach 100 for the best estimation. Furthermore, a good estimator gives consistent, efficient, and unbiased estimates.

## 3 Results and discussion

### 3.1 Gravity

**Fig. 3:**
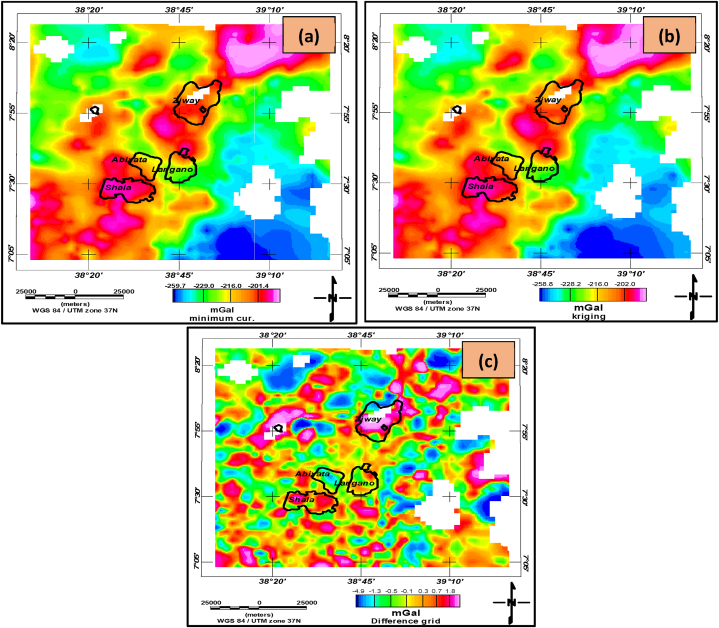
**(a)** Bouguer anomaly map obtained through minimum curvature gridding **(b)** Bouguer anomaly map obtained through kriging interpolant **(c)** the difference grid map.

Preprocessing and Exploratory Data Analysis (EDA) are the first step in any data analysis. They are used to identify the general patterns in the data and help to refine and generate questions. Therefore, the computed complete Bouguer anomaly data were gridded and mapped using minimum curvature (Fig. 3(a)) and kriging interpolants (Fig. 3(b)). The difference anomaly grid (Fig. 3(c)) was obtained by subtracting the two grids (Fig. 3 (a) and (b)). From the difference anomaly grid, it is evident that the two interpolants did not interpolate the Bouguer gravity anomaly data equally.

**Fig. 4:**
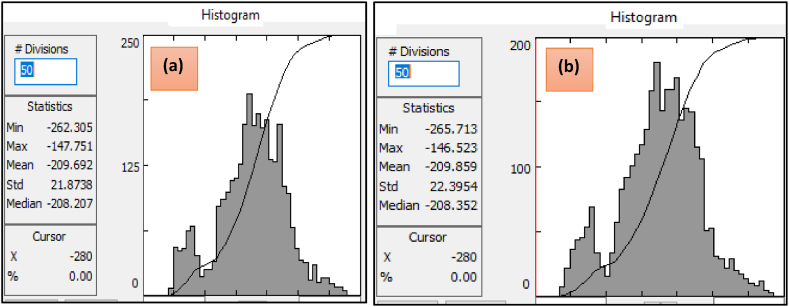
Grid statistics (in mGal) and histogram of Bouguer anomaly gridded through minimum curvature (a) and kriging interpolator (b).

To further examine the difference in the two interpolants, a histogram was created to graphically represent the frequency distributions of grids obtained using minimum curvature and kriging interpolants for the observed Bouguer anomaly data (Fig. 4(a) and (b)). These figures demonstrate the presence of variations between the two gridding methods. Furthermore, summary results displayed to the left of the histograms indicate variations in how the two interpolation methods approximate the magnitude of unsampled and missed datasets.

**Fig. 5:**
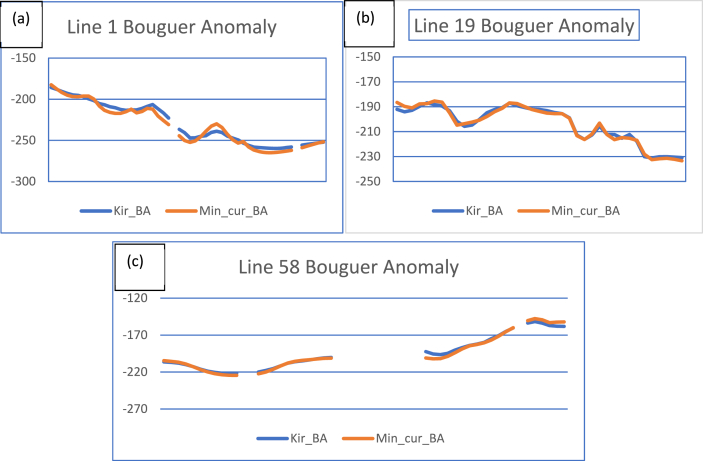
**a)** Bouguer anomaly variability plots along latitude equal to 7.0 decimal degrees **b)** Bouguer anomaly variability plot along latitude equal to 7.74 decimal degrees **c)** Bouguer anomaly variability plot along latitude equal to 8.5 decimal degrees (east-west directions) of the two interpolants grids.

To observe the variability of the interpolants in estimating unsampled gravity data points within the study region, datasets were extracted (sampled) along three lines stretching from west to east at latitudes 7.0 (bottom), 7.74 (middle), and 8.5 (top) decimal degrees respectively. These profile datasets were obtained from Bouguer anomaly grids (Fig. 3a and b) and plotted as shown in Fig. 5 (a), (b), and (c). These graphs show estimations and comparisons using minimum curvature and kriging interpolants. Observations from Fig. 5 (a,b, and c) indicate variabilities between the two interpolants, suggesting that they do not interpolate unsampled data equally.

Following preprocessing and interpretations made on each interpolant, we observe that there is a difference between the two interpolation techniques. Therefore, there is a need to compare and evaluate the interpolation methods by classifying the observed dataset into 90 % being gridded and 10 % left from being gridded. That is, out of the 3012 datasets, 10 % were randomly selected and left from being gridded and the remaining 90 % were gridded using the two interpolants, minimum curvature gridding, and ordinary kriging methods.

**Fig. 6:**
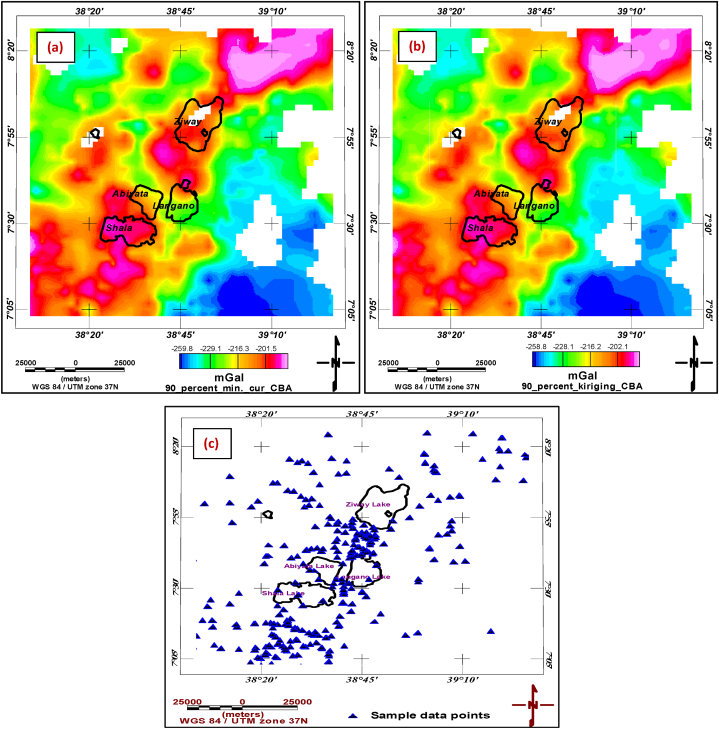
90 % of Bouguer anomaly datasets gridded using (a) minimum curvature gridding and (b) kriging interpolation and (c) the plot of withheld datasets locations (10 % of the overall datasets).

Fig. 6(a)and (b) show the Bouguer anomalies gridded through minimum curvature and kriging interpolators. The maps were obtained after 10 % of randomly sampled test data were withheld. The test dataset's locations (randomly selected) that were not included in the gridding process are shown in Fig. 6(c).

The observed gravity at test data locations (Fig. 6(c)) which were intentionally left from griding (10 % of the data) are now estimated using 90 % data gridded through minimum curvature gridding (Fig. 6(a)) and kriging interpolation (Fig. 6(b)). Sample Observed Bouguer Anomaly (OBA) and estimate made using minimum curvature griding and kriging are shown in Table 1.

**Table 1:** Interpolation techniques that estimate the 10 % Observed Bouguer anomaly (at 301 test data locations).

SN	Lat	Lon	OBA (mGal)	min_cur. Approximate (mGal)	Kriging Approximate (mGal)	OBA_minus_min_cur (mGal)	OBA_minus_kriging (mGal)
1	38.676	7.127	−213.678	−223.322	−222.874	9.644	9.197
2	38.521	7.985	−229.591	−211.458	−211.965	−18.133	−17.626
3	39.176	7.667	−239.404	−225.596	−225.553	−13.807	−13.851
**.**	**.**	**.**	**.**	**.**	**.**	**.**	**.**
**.**	**.**	**.**	**.**	**.**	**.**	**.**	**.**
**.**	**.**	**.**	**.**	**.**	**.**	**.**	**.**
299	38.589	7.73	−225.952	−221.489	−221.802	−4.463	−4.150
300	39.1167	7.8667	−238.083	−225.126	−226.771	−12.957	−11.312
301	38.4551	7.1876	−196.973	−199.186	−198.807	2.213	1.835

To evaluate the performance of minimum curvature and kriging interpolation techniques, we employed two sets of statistics. Table 2 presents both. The first set describes the distribution of values within each dataset independently. These are independent statistics and include measures like average, range, variance, and standard deviation. They are calculated separately for the observed data, the minimum curvature estimates, and the Kriging estimates. The second set of statistics focuses on the comparison between observed and estimated values. These are called comparison statistics and are also shown in Table 2. They include correlation coefficient (r), Root Mean Squared Error (RMSE), and R-squared values. These metrics help us understand how well the minimum curvature and Kriging interpolations replicate the observed data.

**Table 2:** Evaluation metrics Statistics (independent and comparison).

Statistics	OBA (mGal)	BA_min_cur (mGal)	BA_kriging (mGal)	Remark
**Average**	−198.7966	−199.0121	−198.9893	These statistics are independent for each: observed data, minimum curvature interpolation estimate, and Kriging interpolation estimate
**Range**	115.4930	100.3419	99.2267	
**variance**	305.0876	228.1110	222.1337	
**standard D**	17.4668	15.1033	14.9042	
**Correlation**	–	0.8608	0.9986	**Comparison Statistics**: Comparison of observed values with estimates from minimum curvature and kriging interpolation.
**RMSE**	–	8.8767	8.7691	
**R-squared**	–	0.7410	0.7474	

We evaluated the performance of minimum curvature and kriging using a set of 301 test data points not included in the gridding process. Table 2 allows for a direct comparison. The mean value of the test observed data is −198.7966 mGal. Both interpolation methods produced estimates close to this mean, with the kriging estimate (deviation of 0.1927 mGal) and minimum curvature estimate (deviation of 0.2154 mGal). Interestingly, the standard deviations suggest kriging (14.9042 mGal) might capture the data distribution slightly better than minimum curvature (15.1033 mGal).

Table 2 also reveals how well the estimates replicate the observed data. The high correlation coefficients (0.8608 for minimum curvature and a remarkable 0.9986 for kriging) suggest a strong linear relationship. Similarly, the R-squared values (0.7410 for minimum curvature and 0.7474 for kriging) indicate both methods explain a significant portion of the variance in the observed data. Finally, the root mean squared errors (8.8767 for minimum curvature and a slightly lower 8.7691 for kriging) quantify the average magnitude of the errors in the estimates.

Overall, the evaluation metrics in Table 2 suggest ordinary kriging outperforms minimum curvature interpolant in griding gravity data in the study area. This conclusion is supported by both independent statistics (mean and standard deviation) and comparison statistics (correlation, RMSE and R-squared).

**Fig. 7:**
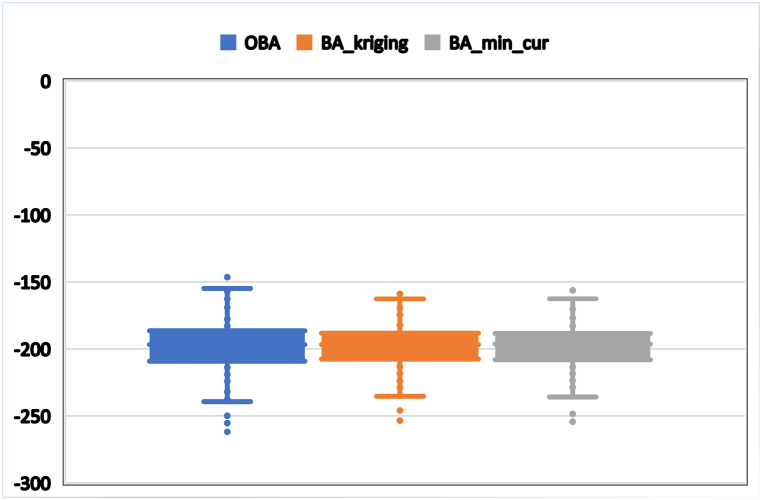
Box and Whisker plot showing true (observed) Bouguer Anomaly (OBA) and predicted anomalies from interpolants, minimum curvature, and krigings.

The boxes in a box and whisker plot (Fig. 7) indicate the interquartile interval where 50 % of the Bouguer gravity datasets are found. It is observed from the Figure that all the datasets are symmetrical. The bottom and top of the boxes indicate the lower and upper quartiles. The horizontal line that splits the box in two is the median of Bouguer gravity anomaly datasets. As shown in Fig. 7, the mean is indicated by a dot on the box plot of the observed anomaly and the estimates. The two lines outside the box that go from the minimum to the lower quartile (the start of the box) and then from the upper quartile (the end of the box) to the maximum are whiskers. The observed anomaly has four outliers; however, the estimate made using the two interpolants each has three outliers indicating the smoothing characteristics of gridding. In comparison, it is observed that the outliers in the case of kriging interpolant were found far from the whiskers end, indicating the good performance of kriging.

**Fig. 8:**
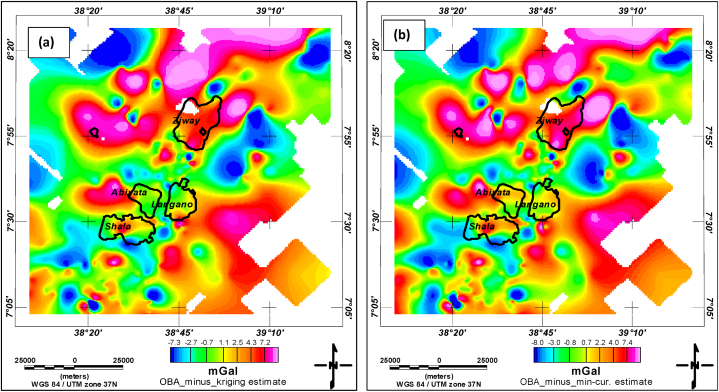
Error plots showing the difference between Observed Bouguer Anomaly (OBA) measurements and a) Kriging estimate and b) minimum curvature estimate.

In addition to the detailed statistics for the estimates (minimum curvature and kriging) and the observed data (presented in Table 1, Table 2, and the box plot), a visual representation of the spatial distribution of errors would be beneficial. This can be achieved by creating two separate two-dimensional (2D) plots with color bars. These plots would show the differences between the observed gravity grid and the estimated grids obtained using minimum curvature and kriging interpolation methods. Fig. 8a and b presents these error grids for the two estimates. It can be read from the error bar of the figure that the values range from −7.3 mGal to 7.2 mGal for the kriging estimate and range from −8.0 mGal to 7.4 mGal for minimum curvature estimates (Fig. 8 a and b). This narrower error range observed in kriging suggests it outperforms minimum curvature interpolation in terms of accuracy in the study region considered in this study.

### 3.2 Elevation

**Fig. 9:**
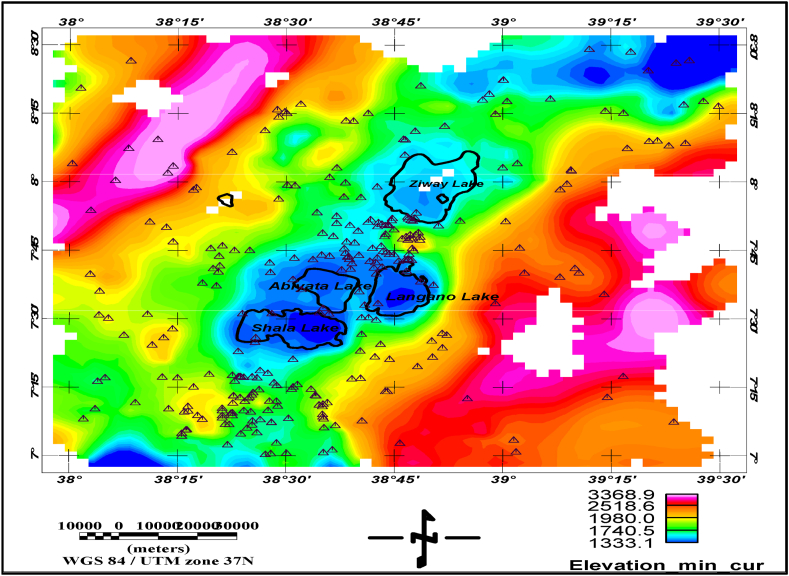
90 % of the elevation datasets were gridded using minimum curvature and 10 % withheld elevation data locations shown in triangular polygons.

Similar preprocessing and Explanatory Data Analysis (EDA), that has been performed for gravity in section 3.1 can be performed for elevation datasets. However, it is not required to document elevation data as the same procedures are followed. Therefore, the elevation dataset was grided with 90 % of the whole dataset and is shown in (Fig. 9). The distribution of the 10 % withheld elevation dataset location that was excluded from the gridding process is shown and overlaid over 90 % gridded elevation datasets (Fig. 9).

The observed 301 elevation data estimates made using minimum curvature are shown in the 4th column and the estimate made using kriging is shown in the fifth column (Table 3). A comparative analysis between the measured data and interpolated values of the observed elevation at the validation dataset has been carried out, revealing various metrics as detailed in Table 4.

**Table 3:** Observed elevation and estimates made using the interpolants.

No.	X	y	Elev. (m)	Elev_min_cur (m)	Elev_Kir. (m)	Difference between	
						Obs. Elev. and minimum curvature (m)	Obs. Elev. and kriging (m)
***1***	*464179.25*	*787767.73*	*2348.00*	*2329.32*	*2325.50*	*18.68*	*22.50*
***2***	*447156.91*	*882626.07*	*1697.00*	*1826.94*	*1824.04*	*−129.94*	*−127.04*
***3***	*519355.35*	*847499.49*	*2428.00*	*2439.68*	*2439.54*	*−11.68*	*−11.54*
	**.**	**.**	**.**	**.**	**.**	**.**	**.**
	**.**	**.**	**.**	**.**	**.**	**.**	**.**
	**.**	**.**	**.**	**.**	**.**	**.**	**.**
***299***	*471668.99*	*868586.16*	*1653.90*	*1667.57*	*1668.60*	*−13.67*	*−14.70*
***300***	*457703.36*	*870365.69*	*1695.00*	*1693.48*	*1693.01*	*1.52*	*1.99*
***301***	*458974.10*	*848718.32*	*1585.00*	*1581.55*	*1582.69*	*3.45*	*2.31*

**Table 4:** Evaluation metrics Statistics (independent and comparison).

Metrics	Observed elevation (m)	Minimum curvature (m)	Kriging (m)	Remark
*Average*	*1856.252*	*1860.696*	*1860.06*	These statistics are independent for each: observed data, minimum curvature interpolation estimate, and Kriging interpolation estimate
*Maximum*	*3298.8*	*3257.951*	*3176.57*	
*Minimum*	*1316*	*1351.738*	*1350.11*	
*Range*	*1982.8*	*1906.213*	*1826.46*	
*Variance*	*88575.322*	*85078.307*	*83052.7*	
*standard D.*	*297.6161*	*291.682*	*288.189*	
*Correlation*	***-***	*0.981*	*0.981*	**Comparison Statistics**: Comparison of observed values with estimates from minimum curvature and kriging interpolation.
*R-squared*	***-***	*0.963*	*0.962*	
*RMSE*	***-***	*57.672*	*58.348*	

The two interpolants do not have the same values. To determine the more suitable interpolants for gridding elevation datasets, various performance metrics were applied. The mean value for the observed data is 1856.2520 m, the minimum curvature griding estimate is 1860.6956 m, and the kriging estimate is 1860.0547 m. Deviation of average values from the observed mean are −4.4436 and −3.8027 respectively for minimum curvature and kriging interpolants. Standard deviation from the mean respectively for Observed, estimate using minimum curvature and kriging are 297.6161 m, 291.6819 m, and 288.1886 m. The Pearson correlation coefficient is given as 0.9811 for minimum curvature and 0.9807 for kriging. The R-squared value for minimum curvature is 0.9625 and for kriging is 0.9617. The RMSE for minimum curvature is 57.6717 and that of kriging estimate is 58.3478. Accordingly, the mean and variance (standard deviation) measures tend to relatively support the performance of kriging interpolants. However, most metrics, such as correlation, RMSE, and R-squared indicate a consistently good performance of minimum curvature interpolations.

**Fig. 10:**
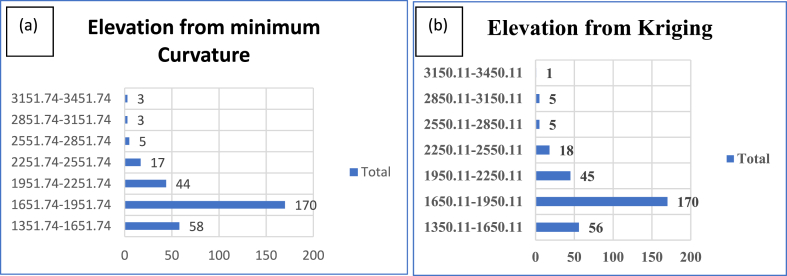
Class intervals in the vertical axis with frequency in the horizontal axis for observed elevation approximation using minimum curvature gridding (a) and predicted using kriging interpolant (b).

Fig. 10 (a and b) show plots showing class intervals in the vertical axis with frequencies in the horizontal axis for the two gridding methods. These interpolation techniques estimate the datasets in each class interval approximately equally well.

## 4 Conclusion and recommendation

In summary, gridding follows after data collection and data preprocessing in the analysis of potential field geophysics. In each data collection, pre-processing, analysis, and discussion, there is a potential for introducing uncertainty. This uncertainty should be either eliminated or minimized at each step. Generally, it is essential to use the appropriate instrument for data collection, the right data collector, and the proper method for preprocessing and analysis for better information extraction about the subsurface. This is especially crucial for geophysical data, particularly gravity anomaly data which is closely related to subsurface geology. The primary focus of this research was the selection of an accurate gridding interpolator method for better estimation of the gravity anomalies caused by subsurface sources. The analysis conducted identified the kriging interpolator as potentially more precise for gravity data analysis compared to minimum curvature. The superior performance of kriging interpolation in this research aligns with the findings from other research locations, as noted in Kamguia et al. [24].

### 4.1 Conclusion

Spatial interpolators are commonly used in many studies to create surface data intended to best represent empirical reality based on a set of sampled points. In this research, the interpolation techniques used were minimum curvature gridding and ordinary kriging. The methodology involves setting aside 10 % of the 3012 data points that remain ungridded. The remaining 90 % of the total datasets were gridded using these interpolants, and these grids were then used as a database for testing the performance of the interpolants against the 10 % of withheld datasets. The result from interpolation techniques must be assessed for accuracy. The various metrics used showed that kriging methods performed better than minimum curvature for gravity data in the study area. Minimum curvature with the least significance performed better than kriging for elevation datasets. Generally, a method that fits well with gravity data can be unsuited for elevation datasets for the study area, although additional tests are needed. Furthermore, there is a need to test the available interpolation methods to select the right gridding techniques.

### 4.2 Recommendation

This research found ordinary kriging to be the most effective method for interpolating Bouguer anomaly data compared to minimum curvature. However, for future studies, a more comprehensive analysis is recommended. This would involve comparing different kriging methods with various input variogram models, along with exploring other interpolation techniques like Inverse Distance Weighting, Bi-directional gridding, and spline functions. Since there's no universally perfect interpolation method, testing their accuracy on the specific data is crucial before final selection.

## Funding statement

No funding was received to assist with the preparation of this manuscript.

## Availability of data and material

The datasets are available from the corresponding author.

## CRediT authorship contribution statement

**Hailemichael Kebede:** Writing – review & editing, Writing – original draft, Visualization, Validation, Supervision, Software, Resources, Project administration, Methodology, Investigation, Funding acquisition, Formal analysis, Data curation, Conceptualization. **Zelalem Demissie:** Writing – review & editing. **Habte Tadesse:** Writing – review & editing. **Addis Eshetu:** Writing – review & editing.

## Declaration of competing interest

The authors declare that they have no known competing financial interests or personal relationships that could have appeared to influence the work reported in this paper.
